# Successful Abdominal Wall Reconstruction Using a Reinforced Tissue Matrix After Severe Pancreatitis and Abdominal Compartment Syndrome: A Case Report

**DOI:** 10.7759/cureus.92698

**Published:** 2025-09-19

**Authors:** David A Baer, James R DeLine

**Affiliations:** 1 General and Endocrine Surgery, AdventHealth Porter, Denver, USA

**Keywords:** abdominal compartment syndrome, abdominal wall reconstruction, complex abdominal defect management, delayed abdominal wall closure, ecm, extracellular matrix, negative pressure wound therapy, npwt, reinforced tissue matrix, severe pancreatitis

## Abstract

Abdominal wall reconstruction following severe abdominal pathology presents a significant surgical challenge. The management of an open abdomen, coupled with the need for delayed closure, often requires advanced techniques to restore abdominal wall integrity, minimize complications, and optimize patient outcomes. This case report describes the successful management of a complex abdominal wall defect following severe pancreatitis and abdominal compartment syndrome in a 48-year-old male patient with a history of alcohol use disorder. After initial decompressive laparotomy and lack of progress with temporary abdominal closure systems, the patient underwent a second procedure at eight weeks to replace the closure system with a reinforced tissue matrix (RTM; OviTex^®^, TELA Bio, Malvern, PA) combined with negative pressure wound therapy (NPWT). Early histopathological analysis of the RTM revealed favorable tissue integration with early remodeling and minimal inflammation. Over the next eight weeks, progressive fascial closure was achieved. Epithelialization reached nearly 100% by 43 weeks post-RTM placement. The final abdominal wall reconstruction at 44 weeks was deemed successful, with satisfactory functional and cosmetic outcomes. This case highlights the effectiveness of combining an RTM and NPWT in the management of complex abdominal wall defects, providing a promising approach for restoring abdominal integrity in high-risk patients. Further studies are needed to assess the long-term outcomes and broader applicability of these techniques.

## Introduction

Abdominal wound closure following laparotomy, particularly in the setting of open abdomen management, remains a significant surgical challenge [1]. Situations such as abdominal compartment syndrome, severe intra-abdominal sepsis, trauma, and necrotizing pancreatitis may necessitate temporary abdominal closure (TAC) to decompress the abdominal cavity, control ongoing contamination, or allow for staged re-exploration [2,3]. However, achieving delayed primary closure can be difficult due to progressive loss of domain, fascial retraction, tissue edema, contamination, or complex wound healing dynamics [4,5]. The risk of wound complications, including fistula formation, infection, and hernia development, is substantial, and long-term functional and aesthetic outcomes are highly variable [6].

A range of adjunctive technologies and techniques has been developed to facilitate the delayed closure of the abdominal wall. These include negative pressure wound therapy (NPWT), biologic and synthetic mesh implants, dynamic closure systems, and component separation techniques [7-10]. Natural extracellular matrix (ECM)-based implants have shown promise in promoting granulation tissue formation and enabling tissue integration, particularly in contaminated or complex fields where synthetic mesh may not be ideal [11-15]. This case report presents a prolonged and multifaceted abdominal wall reconstruction following decompressive laparotomy for abdominal compartment syndrome, highlighting the use of a reinforced tissue matrix (RTM) to achieve successful long-term closure in a challenging clinical scenario.

## Case presentation

**Figure 1:**
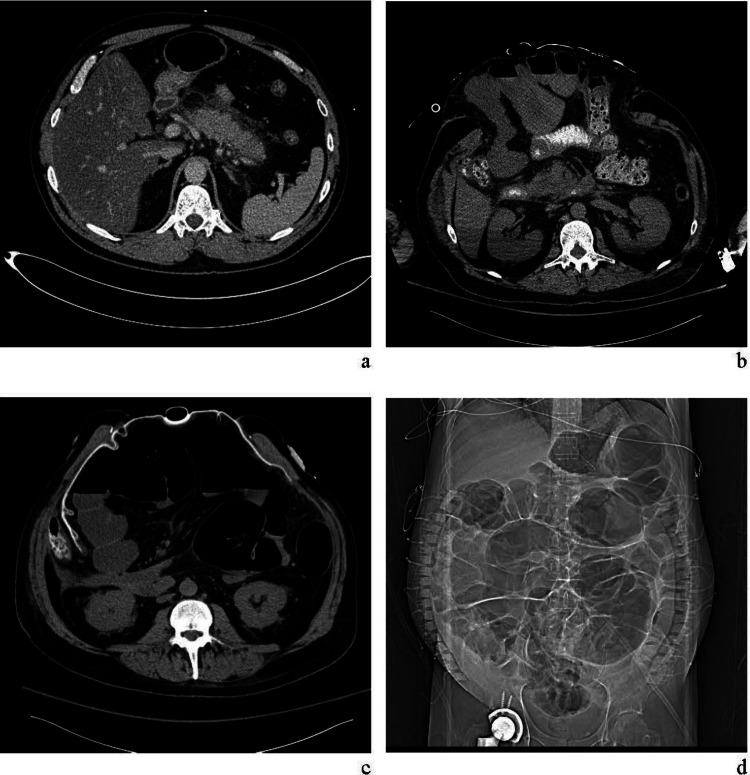
(a) Initial CT scan showing pancreatitis; (b) CT scan showing persistent edema and abdomen distension; (c) CT scan showing persistent ileus and edema and ABThera™; (d) abdominal x-ray showing ileus and ABRA® bands. CT: Computed tomography

A 48-year-old male patient with a history of alcohol use disorder presented to the emergency room with abdominal pain, nausea, and vomiting following a recent episode of heavy episodic drinking. He was subsequently diagnosed with acute pancreatitis (Figure 1a), which rapidly progressed to severe pancreatitis complicated by acute kidney injury and respiratory failure requiring intubation. By the following day, he had developed a distended, tense abdomen with elevated bladder pressure, raising concerns for abdominal compartment syndrome. 

A decompressive laparotomy was performed to decompress the abdominal cavity, yielding 2500 ml of inflammatory intra-peritoneal fluid. After confirming the absence of non-viable organs or tissues requiring resection, a temporary abdominal closure system (ABThera™ abdominal dressing; 3M, St. Paul, MN) was applied circumferentially (Figure 1b,c) and NPWT was initiated to facilitate fluid removal, reduce edema, and promote primary fascial closure over the following eight weeks. During this period, a dynamic wound closure system (ABRA®; Southmedic Inc., Barrie, Ontario, CA), consisting of button anchors and tensioned elastomers designed to gradually approximate the fascial and skin edges was also utilized (Figure 1d). 

**Figure 2:**
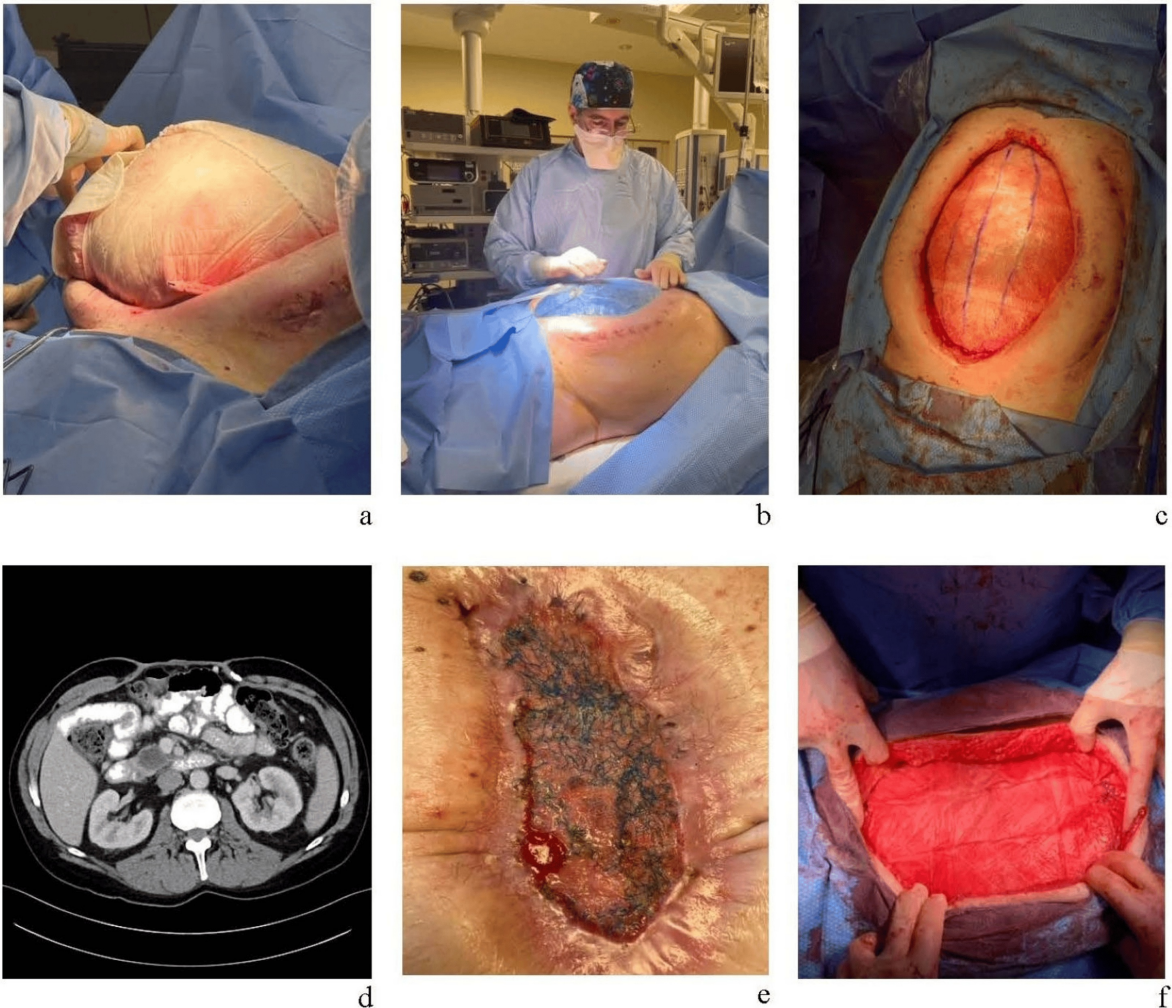
(a) Initial placement of OviTex® RTM. Three large OviTex® 2S RTMs sutured together; (b) Approximately two weeks post-RTM placement, significant decompression of the abdominal cavity can be seen. (c) After removal of previously placed OviTex® RTM and placement of a new OviTex® 1S RTM. The purple lines indicate midline and how close the wound edges could be approximated on either side. (d) CT scan showing ~14 cm diastasis of rectus muscles. (e) Granulation of RTM from intra-abdominal side. Note small bowel fistula in left lower aspect of the image. (f) Approximately 10 months post-initial RTM placement, showing the final RTM placement prior to complete closure of the defect. RTM: Reinforced tissue matrix; CT: computed tomography. Dr. David Baer is the surgeon performing the surgery as seen in figure (b).

After nine weeks, the abdominal contents remained markedly distended, with a mild ileus and persistent hypoalbuminemia despite total parenteral nutrition (TPN). The wound showed minimal progression toward closure. Given the lack of progress with the temporary closure devices, the continued inflammation, and the persistent abdominal wall distension, it was decided to remove the ABThera and ABRA devices and attempt a more definitive closure using a reinforced tissue matrix (RTM; OviTex®, TELA Bio, Malvern, PA). It was hypothesized that the protracted inflammatory response may have been exacerbated by the synthetic materials in the ABThera and ABRA systems, and that transitioning to a biologic material would help mitigate the inflammatory burden and promote resolution of the ileus, thus facilitating better integration with the host tissue and promoting a more effective abdominal wall reconstruction. After removal of the ABThera and ABRA devices, three large pieces of OviTex 2S, including 25x40cm, 25x30cm, and 20x20cm pieces, were sewn together and placed to bridge the abdominal wall gap, then secured to the fascial edges (Figure 2a). NPWT, consisting of a wound vacuum-assisted closure (VAC) system, was applied to maintain RTM hydration. Vaseline-impregnated gauze was placed over the RTM (OviTex®, TELA Bio, Malvern, PA) and covered with polyurethane foam. The wound VAC and foam were routinely replaced throughout the recovery period.

Decompression was achieved one week after RTM placement. The RTM remained intact with no evidence of infection. An additional 10x12cm OviTex 1S RTM was applied to reinforce a thinned area in the lower right quadrant. 

At two weeks post-RTM placement (Figure 2b), a midline portion of the RTM (approximately two inches on each side) was excised and carefully dissected from the underlying granulated tissue. This step was part of a planned, staged approach to gradually approximate the fascial edges without introducing undue tension. The goal was to remove a central strip of the RTM incrementally to facilitate progressive medial advancement of the wound edges and ultimately achieve primary fascial closure. The resulting wound edges were carefully reapproximated without significant tension to prevent separation of the RTM from the wound margins, achieving approximately 50% reduction in wound width. By taking this approach, the remaining RTM was allowed to further integrate in as the patient continued to recover from his injuries.

At three weeks post-RTM placement, the OviTex was replaced. The superficial portions of the existing RTM and the adherent omentum were carefully dissected, exposing the abdominal cavity, which exhibited minimal adhesions. There was no gross evidence of infection or abscess. All residual OviTex material was removed and replaced with a single 20x40cm OviTex 1S piece to cover the previous granulation tissue and the exposed bowel and omentum (Figure 2c). The new approximation reduced the wound width to 9 cm. At this point, the wound measured 30 cm in length (cephalad to caudal).

Overall granulation continued to progress, reaching approximately 80%-85% by nine weeks post-RTM placement. An abdominal CT measured the wound at 8.5 cm in medial lateral length and 10 cm cranial to caudal (Figure 2d). A small bowel fistula was identified in the lower right margin of the RTM (Figure 2e), which was repaired during the subsequent abdominal wall reconstruction procedure. By approximately 18 weeks post-RTM placement, the RTM was nearly 100% granulated with epithelialization at the wound margins. Epithelialization continued through the recovery period, reaching 75%-80% surface coverage by 39 weeks and nearly 100% by 43 weeks post-RTM placement (Figure 2f). During this time, the underlying RTM continued to remodel and incorporate into the underlying tissue, as evidenced by histological analysis of samples obtained at the time of abdominal wall reconstruction as described below.

**Figure 3:**
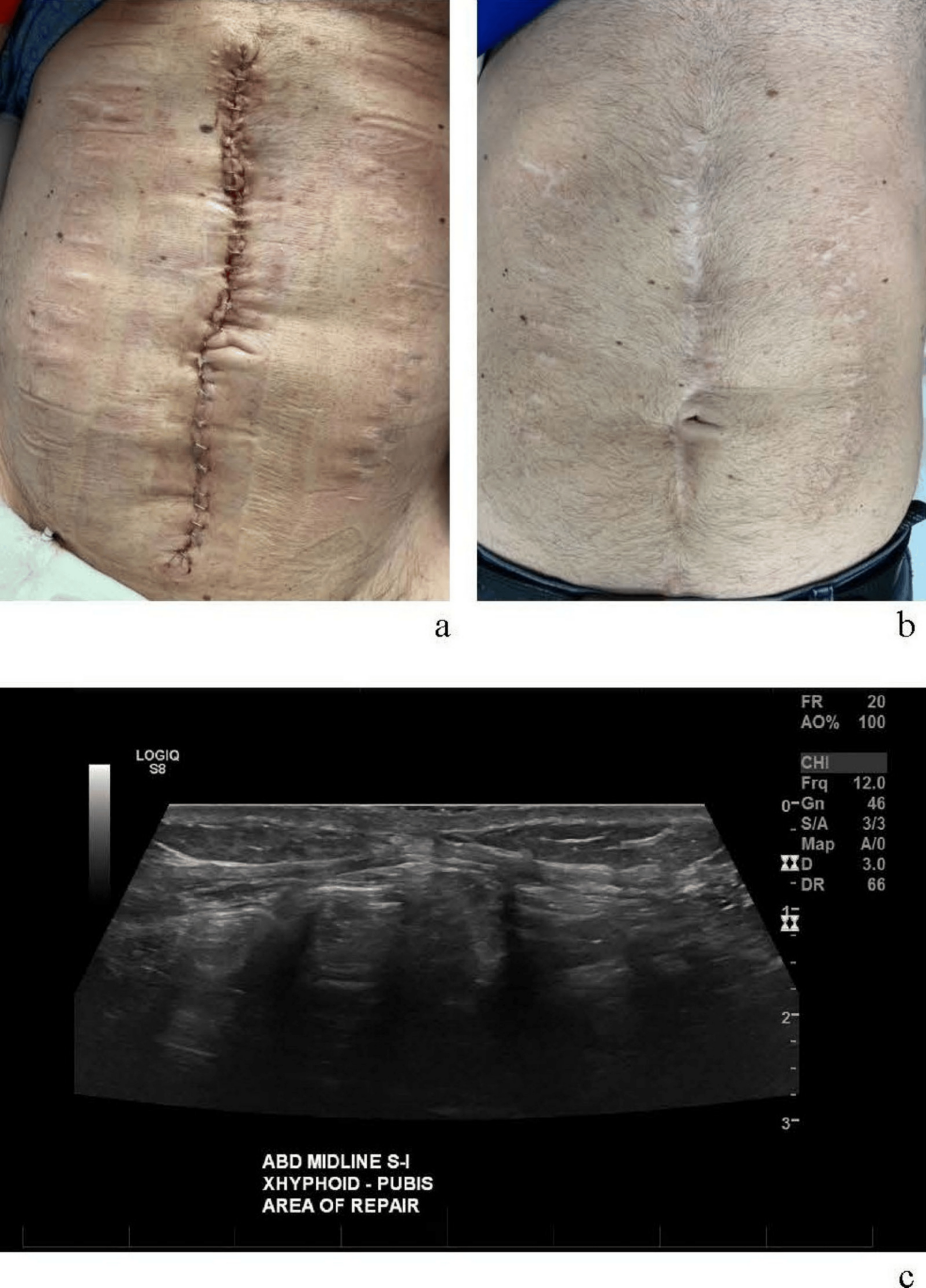
Abdominal reconstruction surgery: (a) Immediately post-op; (b) 20 months post-op; (c) 20-month post-op ultrasound, showing re-approximation of rectus muscles.

At 44 weeks, an abdominal wall reconstruction was performed (Figure 3). During the procedure, the fistula was closed via stapling. The skin was mobilized off the fascial surface circumferentially and the fascia was closed. A 2x2 cm area of dystrophic calcification in the cephalad area of the defect, likely related to scar formation near the disrupted periosteum at the inferior edge of the xiphoid, was observed. This area required excision, and the residual defect was bridged and reinforced with an onlay of OviTex 2S (25x30cm), sewing it to the fascial edges of the wound and around the defect edge to prevent herniation as well. Subcutaneous tissue was reapproximated along the midline and the skin edges were then reapproximated with staples.

**Figure 4:**
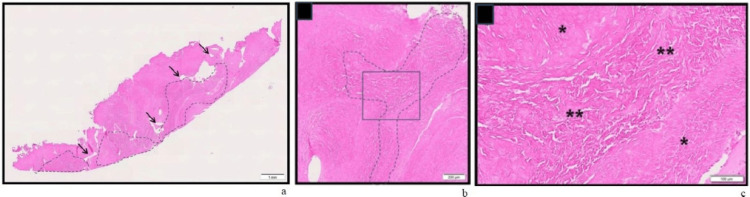
(a) Low magnification view showing the RTM implant (dashed area), polypropylene filament tracts (arrows), and regional collagenous tissue. (b) Intermediate magnification view of boxed area showing focus of the RTM (dashed line) incorporated by surrounding tissue with no inflammatory response present. (c) High magnification view of boxed area showing transition to mature host collagenous tissue (*) infiltrating into collagenous implant (**) indicative of integration and remodeling. RTM: Reinforced tissue matrix

Histological evaluation was performed on samples obtained from the OviTex 1S RTM at the time of the abdominal reconstruction procedure (Figure 4). Paraffin sections were prepared and stained with hematoxylin and eosin (HE) and Masson's trichrome (MT). The findings were consistent with favorable integration of the RTM, demonstrating remodeling, although this could not be confirmed due to the absence of a baseline control. There were no adverse responses noted. Extensive incorporation of the RTM by the host tissue was observed, with tissue extending through the interstitial spaces of the material. This included mature, paucicellular collagen with fine capillary presence. Only a few areas of the RTM exhibited no significant tissue infiltration. Additionally, the inflammatory response was minimal to absent, with only rare, localized lymphohistiocytic infiltrates. These findings suggest a healthy tissue ingrowth, rather than scarring.

The abdominal wall reconstruction procedure was successful, with restoration of abdominal wall integrity and no intra- or post-operative complications. The patient tolerated the procedure well and recovered without evidence of infection, dehiscence, or fistula recurrence. At long-term follow-up (approximately 12-, 18-, and 30-month follow-up), the abdominal wall remained stable, with satisfactory cosmetic and functional outcomes. The patient was able to resume a healthy, active lifestyle. He reported engaging in regular physical activity, including playing basketball and golf, and has maintained sobriety since the procedure. An ultrasound at approximately 20 months post-op was obtained, showing no recurrent hernia/defect below the sternum.

## Discussion

Abdominal wall reconstruction following decompressive laparotomy remains a complex and challenging surgical procedure, particularly in patients with extensive abdominal compartment syndrome, organ failure, or contamination. The management of these patients requires a multidisciplinary approach to optimize outcomes, minimize complications, and restore abdominal wall integrity [16]. In this case, the use of an RTM (OviTex) provided an effective strategy for managing a delayed abdominal wall closure after a period of open abdomen management due to severe pancreatitis complicated by abdominal compartment syndrome.

Delayed primary closure after abdominal decompression is often complicated by factors such as loss of abdominal domain, fascial retraction, contamination, and compromised tissue viability [17]. Several adjunctive techniques, such as NPWT and use of RTMs, have emerged as important tools in addressing these challenges [8]. NPWT has been shown to promote granulation tissue formation and reduce wound size by improving perfusion and minimizing edema [18,19]. In this case, NPWT was employed to enhance granulation and facilitate eventual fascial closure. The use of OviTex RTM provided additional support, promoting tissue integration and enabling the gradual restoration of abdominal wall continuity.

The management of small bowel fistula formation, as observed in this case, is a common complication in patients undergoing open abdomen management [20]. Early identification and surgical intervention are critical in preventing further complications such as peritonitis or sepsis. In our case, the fistula was successfully repaired during the abdominal wall reconstruction procedure, which underscores the importance of addressing such complications during the reconstruction phase.

This case highlights the importance of individualized treatment strategies in managing complex abdominal wall defects. The combination of an RTM and NPWT in this patient resulted in successful abdominal wall reconstruction, with excellent functional and cosmetic outcomes. It also highlights the evolving role of advanced wound management techniques in improving patient outcomes following complex abdominal surgeries.

While the use of an RTM has shown promise in this case, additional long-term follow-up is necessary to fully assess the risk of recurrent hernias, adhesions, and other sequelae in this complex patient population. Additionally, as a single-patient observation, this report is inherently limited in its generalizability of the findings and precludes definitive conclusions regarding efficacy. The absence of a control group, potential for bias, and possible influence of patient-specific factors also restrict broader interpretation. Nonetheless, such reports provide valuable insights into complex clinical scenarios and can inform future studies.

## Conclusions

This case underscores the successful use of a multidisciplinary approach to manage a complex abdominal wall defect following severe pancreatitis and abdominal compartment syndrome. The combination of a reinforced tissue matrix (OviTex RTM) and NPWT facilitated a gradual and controlled reconstruction of the abdominal wall, resulting in favorable functional and aesthetic outcomes. Histopathological analysis demonstrated favorable integration with the host tissue, further supporting the efficacy of this RTM in complex abdominal wall reconstructions.

While advanced closure techniques offer significant benefits, they require careful management and long-term follow-up to assess potential complications such as hernia formation, adhesions, and tissue failure. This case highlights the evolving role of reinforced tissue matrices in complex abdominal surgeries and emphasizes the importance of individualized treatment strategies to optimize patient outcomes.
